# Human Pluripotent Stem Cell-Derived Medium Spiny Neuron-like Cells Exhibit Gene Desensitization

**DOI:** 10.3390/cells11091411

**Published:** 2022-04-21

**Authors:** Ryan W. Tam, Albert J. Keung

**Affiliations:** Chemical & Biomolecular Engineering Department, North Carolina State University, Campus Box 7905, Raleigh, NC 27695, USA; rwtam@ncsu.edu

**Keywords:** dopamine, gene desensitization, acute and chronic, RNA-seq, human embryonic stem cell, medium spiny neuron

## Abstract

Gene desensitization in response to a repeated stimulus is a complex phenotype important across homeostatic and disease processes, including addiction, learning, and memory. These complex phenotypes are being characterized and connected to important physiologically relevant functions in rodent systems but are difficult to capture in human models where even acute responses to important neurotransmitters are understudied. Here through transcriptomic analysis, we map the dynamic responses of human stem cell-derived medium spiny neuron-like cells (hMSN-like cells) to dopamine. Furthermore, we show that these human neurons can reflect and capture cellular desensitization to chronic versus acute administration of dopamine. These human cells are further able to capture complex receptor crosstalk in response to the pharmacological perturbations of distinct dopamine receptor subtypes. This study demonstrates the potential utility and remaining challenges of using human stem cell-derived neurons to capture and study the complex dynamic mechanisms of the brain.

## 1. Introduction

Complex cellular programs (e.g., transcriptional networks, epigenetics) respond to spatiotemporally diverse extracellular perturbations. These responses, including gene desensitization to repeated stimuli, act as homeostatic mechanisms to stress and are important for processes such as addiction [1,2], learning, and memory [3]. These phenotypes have been primarily studied in rodents but sparsely investigated in human models [4,5,6,7]. However, there are likely species-specific differences between humans and other animals and these differences may be relevant for the success of therapeutic strategies [8,9]. Therefore, it is important to assess complex cellular responses in ethical human models.

A salient example of gene desensitization is the complex response of medium spiny neurons, the primary cell type of the striatum [1,2], to the chronic administration of substances of abuse. In particular, cocaine and other drugs of abuse modulate the timing and levels of dopamine that presynaptic dopaminergic neurons [10,11] expose postsynaptic MSNs to, with postsynaptic transcriptomic and functional effects on MSNs [12,13,14,15]. More generally, MSNs serve as the main input and projection cells of the basal ganglia and thus play a role in the generation of behaviors [16]. Thus, in addition to human MSNs (hMSNs) serving as a potential model to study gene desensitization in human cells, they may also yield insights into the mechanisms underlying aspects of human behavior.

Protocols have previously been developed to differentiate human pluripotent stem cells (hPSCs) into neurons expressing stereotypical markers of striatal MSNs [17,18,19,20,21,22]. However, these studies generally aimed to model disorders such as Huntington’s Disease and genetic forms of Parkinson’s Disease and their relatively static phenotypes. In this work, we evaluate the dynamic transcriptomic responses of hMSN-like cells to a range of acute and chronic dopamine exposures, identify experimental concentrations and timings correlating with specific types of MSN responses including desensitization, and discuss the advantages and challenges facing this model system illuminated by the results. This work advocates for the continued expansion of work leveraging human models to investigate complex dynamic cellular responses [4,15].

## 2. Materials and Methods

### 2.1. Cell Cultures

hMSN-like cells were obtained following previously described protocols [17]. Feeder-independent cell lines were H9 and H1 hESCs (WA09 and WA01; WiCell, Madison, WI, USA). Cells were maintained in 6-well tissue culture dishes (Greiner Bio-One, Alphen aan den Rijn, NL) coated with 0.5 µg/mL reduced growth factor Matrigel solution (Corning, Durham, NC, USA) in E8 medium (Stemcell Technologies, Cambridge, MA, USA) and passaged using standard protocols. Cells were grown to ~75–80% confluency prior to differentiation. On days in vitro (DIV) 0 of differentiation, E8 medium was removed, cells were washed with 1X PBS (Gibco, Waltham, MA, USA), and media switched to DMEM-F12/Neurobasal media (2:1) (Gibco) supplemented with N2 (Gibco) and B27 minus Vitamin A (Gibco) (together referred to as N2B27). From DIV 0 to 4, cultures were supplemented with SB431542 (10 µM in 95% EtOH; Selleck Chemicals, Houston, TX, USA), LDN-193189 (100 nM in DMSO; BioVision, Waltham, MA, USA), and dorsomorphin (200 nM; BioVision). Media was changed every day. From DIV 5 to 8, cultures were supplemented with just LDN-193189 and dorsomorphin. Media was changed every day. On DIV 9, cultures were washed with 1X PBS, and media switched to N2B27 supplemented with activin A (25 ng/mL in 4 mM HCl; R&D, Minneapolis, MN, USA). Cells were then lifted using cell scrapers (Greiner Bio-One) since EDTA treatment drastically reduced survivability, pipetted up and down 1 time with a 5 mL serological pipette, and replated with 5.21 × 10^5^ cells/cm^2^ onto Matrigel-coated 6-well plates. Half-media was changed the next day and then every other day afterward. On DIV 18, cultures were passaged using 0.5 mM EDTA for 2 min at 37 °C, as we no longer observed issues with survivability with EDTA treatment, and pipetted up and down 10–15 times using P1000 mechanical pipette then replated onto Poly-L-Ornithine (15 µg/mL in water; Sigma) and Laminin (5 µg/mL in PBS; Corning) coated 24-well plates (Greiner Bio-One) at 2.11 × 10^5^ cells/cm^2^. Half-media was changed the next day and then every other day afterward. From DIV 20 to 24, media was switched to N2B27 with Vitamin A (Gibco). On day 25 for analysis, BDNF and GDNF (10 ng/mL in 0.1% BSA (*w*/*v*); Peprotech) were added to aid neuronal maturation and survival (referred to as Maturation Medium). Cells were lifted, similar to the DIV18 passage, on DIV 30 to 35 and all cells were replated onto Poly-L-Ornithine (2 µg/cm^2^) and Laminin (1 µg/cm^2^) coated 24-well plates to maintain neuron attachment. Cells were maintained in a humid incubator at 37 °C with 5% CO_2_.

### 2.2. Dosing Experiments

To perform acute incubations, hMSN-like cells were grown to DIV45, half-media was removed, replaced with dopamine hydrochloride (10 nM–1 mM in 100 µM ascorbic acid; Sigma, St. Louis, MO, USA), quinpirole (1–100 µM; Sigma, St. Louis, MO, USA), or CGS21680 (0.1–10 µM; Sigma, St. Louis, MO, USA), and incubated at 37 °C for 60 min for RNA-seq. Chronic incubation proceeded by removing all culture media, replacing with maturation media, and repeating dopamine incubations for 2, 3, 4, or 5 days. Chronic dopamine time-course doses were time-matched to extract all samples on DIV50. Comparisons of 1 mM and 1 µM dopamine doses were performed on DIV45 for acute dosage and DIV50 for chronic dosage.

### 2.3. Immunocytochemistry

Prior to immunocytochemistry, cultures were grown in 8-well chamber slides (Falcon) to DIV 45. Cultures were fixed in 4% paraformaldehyde for 15 min at room temperature (RT) followed by 3 × 5 min 1X PBS washes. Cultures were then permeabilized and blocked in 0.3% Triton X-100 in PBS (PBST) and 5% (*w*/*v*) normal donkey serum (Jackson Immunoresearch) in PBS for 30 min at RT. Cultures were incubated with primary antibodies (see Table S1) in PBST at RT for 2 h or at 4 °C in a humidity chamber overnight. Cultures were washed by 3 × 10 min PBST washes and incubated with secondary antibodies in PBST for 2 h at RT, and nuclei were stained with DAPI (Invitrogen, Waltham, MA, USA). Slides were mounted using ProLong Antifade Diamond (Thermo Fisher Scientific, Waltham, MA, USA). Secondary antibodies used were donkey Alexa Fluor 546 and 647 conjugates (Invitrogen, 1:250). Images were taken using a Nikon A1R confocal microscope (Nikon Instruments, Tokyo, Japan) using a 40X oil immersion objective. To ‘fill’ the cell to assess morphology, hMSN-like cells were transfected with a pmaxGFP plasmid (Lonza, ThermoFisher Scientific, Melbourne, Australia) using Lipofectamine 3000 Transfection reagent (Thermo Fisher) 72 h before fixation following the manufacturer’s instructions.

### 2.4. Immunostaining Quantification

To quantify FOSB+ and DARPP32+ cell percentages, immunostained slides were imaged using a 40X oil immersion objective for DAPI, FOSB, MAP2, and DARPP32 simultaneously using the 405, 488, 561, and 640 nm lasers. ND2 stacks were imported into FIJI [23], Z-projected for max intensity, converted to grayscale, split into individual channels and background subtracted for each channel individually using a 50-pixel rolling ball radius. Images were thresholded using the “Moments” algorithm with the “Auto” setting and measured for area fraction. Overlap percentages were obtained by combining thresholded FOSB or DARPP32 channels with either DAPI or MAP2 using the Image Calculator function with the “AND” operation, measuring area fraction, and dividing the overlap area fraction by the DAPI or MAP2 area fraction. For FOSB overlap with DARPP32/MAP2, “AND” combined DARPP32 and MAP2 channels were “AND” combined with the thresholded FOSB channel. Error bars were calculated as standard deviation. For each sample, 2–3 independent replicates (*n* = 2–3) in 8-well chamber slides were dosed, fixed, and immunostained.

### 2.5. RNA Extraction and QRT-PCR

For RNA extraction, total RNA was extracted as previously described [24]. In brief, hMSN-like cultures were washed 1 time in PBS. Total RNA was isolated using Direct-zol RNA MicroPrep Kit (Zymo Research) according to the manufacturer’s protocol. RNA samples were collected in 2 mL RNAse-free tubes and chilled on ice throughout the procedure. Total RNA was then used for either QRT-PCR or RNA-seq. cDNA synthesis was performed using 1000 ng of total RNA and the iScript Reverse Transcription Kit (BIO-RAD) according to the manufacturer’s protocol. QRT-PCR reactions were performed using SsoAdvanced™ Universal SYBR^®^ Green Supermix (BIO-RAD) on a BIORAD 384-well machine (CXF384) with custom-designed primers using Primer3 [25] (see Table S2). Analysis of target gene expression along with the reference genes HPRT1 and GUSB was performed using Excel. Data are presented as expression level (2^−ΔΔCt^) relative to HPRT1 and GUSB and normalized to control dose. For each QRT-PCR sample, 3 independent replicates (*n* = 3) in individual 24-well plate wells were collected. Two technical replicates per sample were also included with each QRT-PCR experiment. All QRT-PCR analyses were performed on RNA extracted from DIV 45 hMSN-cells.

### 2.6. RNA-Sequencing and Analysis

Sequencing and preparation of Illumina libraries from total RNA was performed and then sequenced using Illumina Hi-seq 2 × 150 bp (Azenta, Research Triangle Park, NC, USA) and NovaSeq S1 2 × 100 bp (UNC High Throughput Sequencing Facility) for 20–30 million reads per sample. Raw sequencing data are publicly available on the Gene Expression Omnibus (GEO Accession Number: GSE195492).

Raw FASTQ formatted sequence reads were imported into CLC Genomics Workbench (v. 21.0.5 Qiagen, https://digitalinsights.qiagen.com/, accessed on 12 January 2022). Adaptor sequences and bases with low quality were trimmed and reads were mapped to the reference genome (GRCh38.102) using the RNA-seq analysis tool with the default parameters recommended for RNA-seq analysis. Principal component analysis and differential expression analysis were performed using ‘PCA for RNA-seq’ and ‘Create Heat Map for RNA-seq’ toolsets. Heatmaps were generated using Euclidean distance with complete linkage. All genes were displayed. Ingenuity Pathway Analysis (v. 22.0, QIAGEN Inc., https://digitalinsights.qiagen.com/IPA, accessed on 12 January 2022) was used to predict upstream regulators [26]. Functional enrichment analysis was performed using gProfiler to extract KEGG pathways, biological process and molecular function gene ontologies, and transcription factor binding motifs associated with an inputted list of genes [27]. Other plots and analyses were generated using RStudio [28]. Overlaps between gene sets were performed using BioVenn [29] and the Venn Diagram tool from the Bioinformatics & Evolutionary Genomics Department at Ghent University [30]. Log_2_(fold change) heatmaps were generated using pheatmap (v. 1.0.12). Boxplots and Lineplots were plotted using ggplot2 (v. 3.3.5) [31]. Significance was defined as *p* < 0.05. Statistical analyses for RNA-seq were performed in CLC Genomics Workbench (v. 21.0.5 Qiagen, https://digitalinsights.qiagen.com/, accessed on 12 January 2022).

### 2.7. Statistical Analysis

Statistics were performed for QRT-PCR using the one-way ANOVA with Tukey’s post hoc test to test for significance. All analyses were performed using Rstudio. Significance was defined as *p* < 0.05. Statistical analyses for RNA-seq were performed in CLC Genomics Workbench (v. 21.0.5 Qiagen, https://digitalinsights.qiagen.com/, accessed on 13 January 2022). Significance was selected as log_2_(fold change)  ≥ |1| in expression between comparison groups with a threshold false discovery rate (FDR), adjusted *p*-value < 0.05, and max group mean ≥ 1 for acute and chronic dopamine datasets. Significance for all other RNA-seq datasets was selected as (FDR) adjusted *p*-value < 0.05. All sequencing data passed default quality filters for the CLC Genomic Workbench version 21.0.5 RNA-Seq pipeline analysis. Significance for Ingenuity Pathway (v. 22.0, QIAGEN Inc., https://digitalinsights.qiagen.com/IPA, accessed on 13 January 2022) and gProfiler [27] analyses were defined as *p*-value < 0.05 and g:SCS threshold < 0.05, respectively.

## 3. Results

### 3.1. hPSC-Derived Neurons Express Markers of MSNs

**Figure 1:**
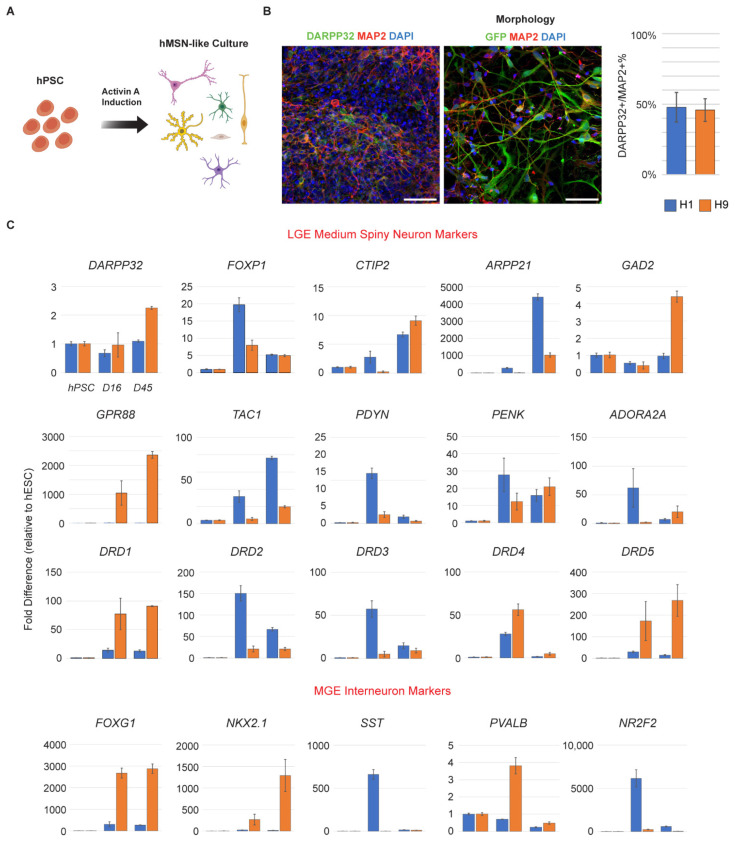
hPSC-derived neurons express markers of MSNs. (**A**) hPSCs are differentiated into hMSN-like cells following an Activin A induction protocol (Arber et al., 2015 [17]), (**B**) immunostained for MAP2+ (red), DARPP32 (green), and DAPI (blue) and quantified for DARPP32+ percentage. GFP-transfected hMSN-like cells were imaged to better highlight cell morphology. Scale bar = 50 µm. (**C**) QRT-PCR of RNA isolated from H1 (blue) and H9 (orange) hPSCs and hPSCs differentiated into hMSN-like cells at DIV 16 (D16) and DIV 45 (D45), for genes of interest. Values were normalized to HPRT1 mRNA levels in the same samples and expressed as normalized fold changes in hMSN-like versus hPSC cells. Values normalized to GUSB are provided in Figure S1. Gene categories are labeled in red. *n* = 3–4 independent replicates & 2 technical replicates. Error bars = Standard error.

We differentiated male (H1) and female (H9) human embryonic stem cells with an Activin A induction protocol (Figure 1A) [17]. After 45 days (DIV 45), hMSN-like cells were immunostained and found to express DARPP32 and MAP2, the stereotypical markers of striatal MSNs and neurons, respectively (Figure 1B, Table S1). Additionally, hMSN-like cells differentiated into neurons that were 47.8% and 45.9% DARPP32+ in H1 and H9, respectively (Figure 1B). QRT-PCR at days 16 and 45 showed that hMSN-like cells also expressed other general markers of lateral ganglionic eminence MSNs including *FOXP1*, *CTIP2*, and *ARPP21* (Figure 1C, Table S2). MSNs are often classified into two main subtypes: dopamine receptor D1-like and D2-like receptor-expressing MSNs [32,33]. We identified the markers of both D1 (i.e., *DRD1*, *TAC1*, *PDYN*) and D2 (i.e., *DRD2*, *PENK*, *A2A*) MSNs. As expected, hMSN-like cells also expressed acetylcholine (e.g., *CHRM1*) and glutamate receptors (e.g., *GRIA1*, *GRIN1*) (Figure S1) [34]. hMSN-like cells also expressed genes characteristic of medial ganglionic eminence interneurons (i.e., *NKX2.1*) (Figure 1C).

### 3.2. hMSN-like Cells Exhibit Dose and Time-Dependent Responses to Dopamine

**Figure 2:**
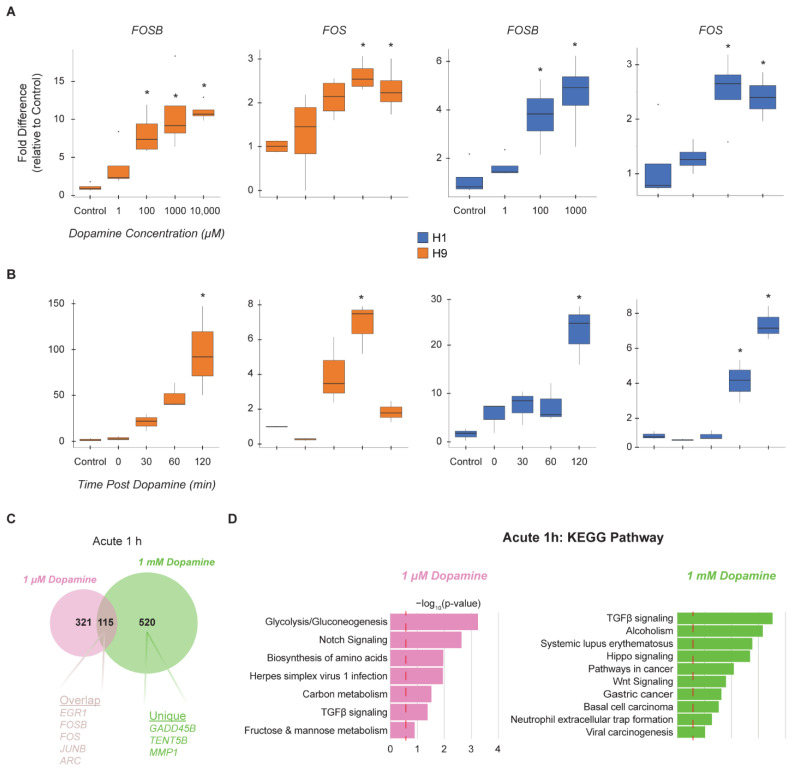
hMSN-like cells exhibit dose and time-dependent responses to dopamine. (**A**) QRT-PCR of RNA isolated from DIV45 H1 and H9 hMSN-like cells 1 h after exposure to different dopamine concentrations (1 µM to 10 mM) and analyzed for *FOSB* and *FOS*. (**B**) QRT-PCR of RNA isolated from DIV 45 H1 and H9 hMSN-like cells 0 to 120 min after exposure to 1 mM dopamine and analyzed for *FOSB* and *FOS*. (**A**,**B**) For QRT-PCR, values were normalized to *GUSB* mRNA levels in the same samples and expressed as a fold change in dopamine versus PBS control cultures. * = *p* < 0.05; One-way ANOVA. *n* = 3–4 independent replicates and 2 technical replicates. (**C**) Venn Diagram showing the number of shared differentially expressed genes (DEG) between DIV45 hMSN-like cells quantified by RNA-seq 1 hour after acute 1 μM and 1 mM dopamine. (**D**) Functional enrichment analysis of RNA-seq data for KEGG pathways of DEGs unique to DIV45 1 μM dopamine dosed (left) and 1 mM dosed (right) hMSN-like cells. Significance is represented by Log_10_-transformed *p*-values. Dotted red line indicates *p*-value of 0.05. (**C**,**D**) DEGs were identified by max group mean ≥ 0.75, FDR *p*-value < 0.05, and Log_2_(Fold Change) > |1|. Differential expression was performed against PBS control group using the Wald test. *n* = 3 independent replicates.

MSNs are expected to respond to dopamine, a key neurotransmitter in the striatum, although it remains unclear what the dose dependency and kinetics of the responses are in hMSN-like models [20]. Therefore, we first characterized the dose-dependent response of hMSN-like cells to dopamine. It is still unclear what concentrations would best mimic the effective local concentrations of dopamine in the synaptic cleft. Estimates of neurotransmitter concentrations vary widely from 1 µM to 1 mM and are heavily dependent on whether measurements were made directly in the cleft or in the extracellular space. Prior work in ex vivo rodent models suggested 100 nM–100 µM and 30–60 min time points would be reasonable to elicit physiologically relevant responses [35,36,37,38,39,40,41]. For example, *FOSB* and *FOS* are immediate early genes (IEG) previously observed to be induced in rodents by a minimum of 100 µM dopamine [40,42]. We, therefore, dosed both H1- and H9-derived DIV45 hMSN-like cells with a range of dopamine concentrations from 1 µM to 10 mM and tracked *FOSB* and *FOS* expression by QRT-PCR after 0 to 120 min (Figure 2A). Gene expression induction was observed at all concentrations and reached maximal expression at a concentration of 1 mM dopamine. We also tracked IEG induction at 0, 30, 60, and 120 min post-addition of 1 mM dopamine and observed that maximal induction was observed between 60 to 120 min (Figure 2B). The *FOSB* and *FOS* measurements indicated that hMSN-like cells respond acutely to dopamine. Additionally, immunostaining revealed FOSB expression in 11.21% of DIV45 H9 DAPI+ and 4.7% of DIV 45 H1 DAPI+ cells after acute dopamine dosage (Figure S2). Having observed greater responses in H9-derived cells, we decided to perform all remaining experiments using H9 hMSN-like cells. We next assessed the transcriptome-wide responses and performed an RNA-seq 1 h after dosing the DIV45 H9 hMSN-like cells using either 1 µM or 1 mM dopamine (Figure 2C,D). The overlap in the DEGs was low between the two concentrations (Figure 2C, Dataset S1); yet, reassuringly, among the DEGs that were common included many genes canonically relevant to substance-use disorders and neuronal stimulation such as *EGR1*, *FOSB*, *FOS*, *JUNB*, and *ARC*. The KEGG pathway analysis suggested some general differences between 1 µM and 1 mM concentrations where the 1 mM dose led to DEGs associated more with the signaling pathways (i.e., TGFβ, Wnt, & Hippo Signaling) (Figure 2D). As expected, the larger 1 mM concentration of dopamine led to a greater response compared to the 1 µM dopamine dose [43,44,45]. Furthermore, the presence of some specific DEGs suggests that the 1 mM concentration may be more informative for studies related to substances of abuse. For example, *GADD45B* (implicated in memory of cocaine reward) [46], *TENT5B* (the top DEG observed in dopamine-dosed rodent MSNs) [40], and *MMP1* (extracellular matrix and synaptic plasticity) [47] were elevated only in the 1 mM dopamine conditions. However, the expression of individual DEGs should not be overinterpreted as they were not individually confirmed by QRT-PCR. Instead, here we focus on collections or sets of related DEGs through ontologies or key phenotypes that reflect neuronal responses.

### 3.3. Chronic Administration of Dopamine Leads to Desensitization of Genes Implicated in Cocaine and Dopamine Responses

**Figure 3:**
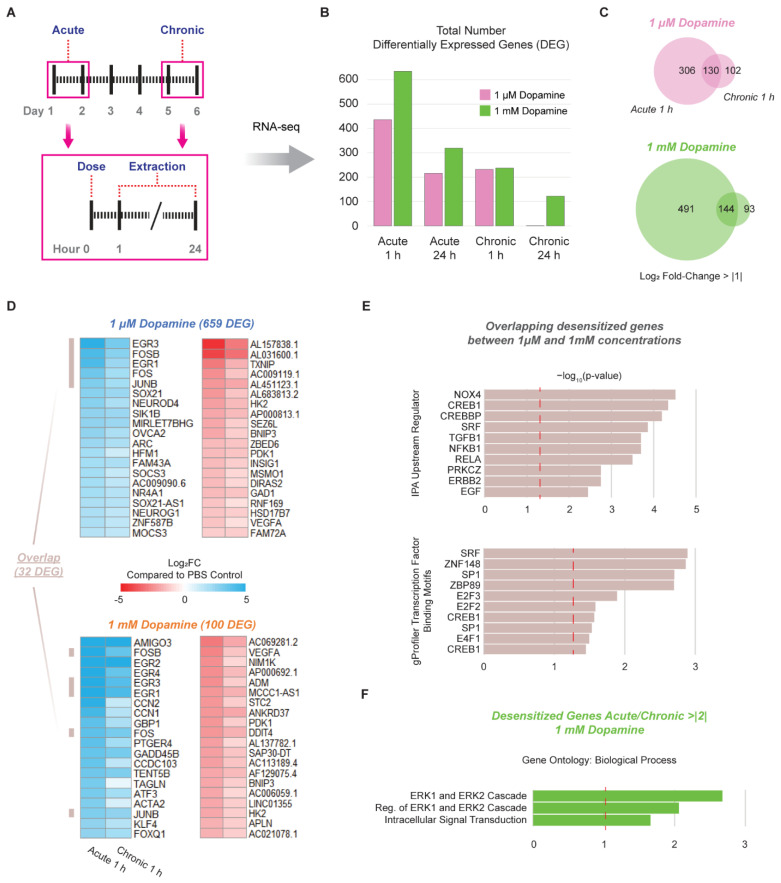
Chronic administration of dopamine leads to desensitization of genes implicated in cocaine and dopamine responses. (**A**) Schematic for isolation of RNA from H9 hMSN-like cells dosed acutely (DIV45) and chronically (DIV50) with dopamine. (**B**) Total number of DEGs from RNA-seq of H9 hMSN-like cells dosed with 1 μM and 1 mM dopamine. (**C**) Venn diagrams showing shared number of DEGs 1 hour after dosage between hMSN-like cells dosed with DIV45 acute and DIV50 chronic dopamine. (**D**) Heatmaps of top 20 desensitized genes for hMSN-like cells exposed to acute and chronic dopamine. Desensitized genes are defined as the ratio of Acute 1 h Log_2_(Fold Change)/Chronic 1 h Log_2_(Fold Change) > |1.1|. Overlapping genes highlighted by tan-colored bars. (**E**) IPA upstream regulators and gProfiler transcription factor regulatory motifs of desensitized genes common between 1 μM and 1 mM dopamine conditions. (**F**) Gene ontology biological processes for highly desensitized genes after chronic 1 mM dopamine, defined when the ratio Acute 1 h Log_2_(Fold Change)/Chronic 1 h Log_2_(Fold Change) > |2|. (**E**,**F**) Significance is represented by Log_10_-transformed *p*-values with dotted red line indicating *p*-value of 0.05. In all cases, data were obtained from RNA-seq of H9 hMSN-like cells. DEGs were identified by max group mean ≥ 0.75, FDR *p*-value < 0.05, and Log_2_(Fold Change) > |1|. Differential expression was performed against PBS control group using the Wald test. *n* = 3 independent replicates.

A key physiological response to substances of abuse is desensitization at the cellular level. The chronic dosage or self-administration of substances of abuse in rodents leads to a unique molecular phenotype known as gene desensitization [1,14,48], in which differentially expressed genes (DEG) reduce in magnitude of expression when compared to a single acute exposure. To assess if this important response also occurs in hMSN-like cells, we acutely (DIV45) or chronically (DIV50) dosed hMSN-like cells with dopamine, extracted RNA at 1 h or 24 h after the last dose, and performed RNA-seq (Figure 3 and Figure S3). As both the 1 µM and 1 mM concentrations elicited gene expression responses but to distinct degrees (Figure 2A) and it remains unclear what concentration is truly physiological, we tested both. Importantly, at both concentrations, chronic dosing led to a dramatic reduction in the number of DEGs compared to single acute doses (Figure 3B). As expected, the number of DEGs decreased when sampling at 24 h versus 1 h after the last dopamine exposure, although a substantial number of DEGs remained (see Dataset S1–S4). To identify whether the same set of genes was responding to both acute and chronic dosing, we compared DEGs across conditions and observed that 50–60% of the chronically dosed hMSN-like cell DEGs were also differentially expressed after acute dosing (Figure 3C). This was true for both concentrations. However, although the overall number of DEGs decreased, this did not mean that individual genes were necessarily desensitizing. Therefore, we narrowed our analysis to identify the desensitized genes. To do this, we ratioed the gene expression changes of acute 1 h hMSN-like cells to chronic 1 h hMSN-like cells and did so for 1 µM and 1 mM dopamine conditions separately. Desensitized genes were defined as those with a ratio > |1.1| as previously defined [1]. In general, the 1 mM dopamine conditions led to a larger number of desensitized genes compared to the 1 µM conditions (Figure 3D; see Dataset S5). However, both concentrations shared overlap in important desensitized genes (Figure 3D). Specifically, genes that were desensitized in both concentrations of dopamine included many immediate early genes (e.g., *FOSB*, *FOS*, *JUNB*) (Figure 3D). Additional overlap was identified through IPA analysis including genes that were associated with the upstream regulators CREB1 and SRF, transcription factors that are necessary for ∆FOSB induction including in response to cocaine; and NFκB1/RELA, which are involved in regulating the reward properties of drugs of abuse (Figure 3E) [49,50]. Interestingly, we also noted an increase of FOSB+ cells after chronic dopamine dosage (DIV50) compared to acute dosage (DIV45) for both H1 and H9 hMSN-like cells (Figure S2). This may be indicative of an increase in the ∆FOSB concentration after chronic dosage. We also performed a regulatory motif analysis of desensitized DEGs present for both concentrations and found that the motifs for SRF and CREB1 were enriched, which was similar to the IPA analysis results. In addition, the motifs for E2F3 binding, a transcription factor that regulates cocaine-induced locomotor and place-conditioning behavior, were also enriched (Figure 3E) [51]. To identify differences in the nature of the desensitized genes between the 1 mM and 1 µM concentrations, we also analyzed genes that were highly desensitized to a 1 mM dopamine dose which we defined as beyond the maximum level of desensitization observed after a 1 µM dose (Acute 1 h to Chronic 1 h ratio > |2|). Genes dosed with 1 mM dopamine with an Acute 1 h to Chronic 1 h ratio |>| 2 were enriched for biological process GO related to ERK1/2 cascades (Figure 3F). Thus, differences in desensitization between the 1 mM and 1 µM concentrations were in large part acting through ERK signaling.

### 3.4. Time Course of Chronic Dopamine Administration Reveals Peak in DEGs at Day 3 and Desensitization at Day 5

**Figure 4:**
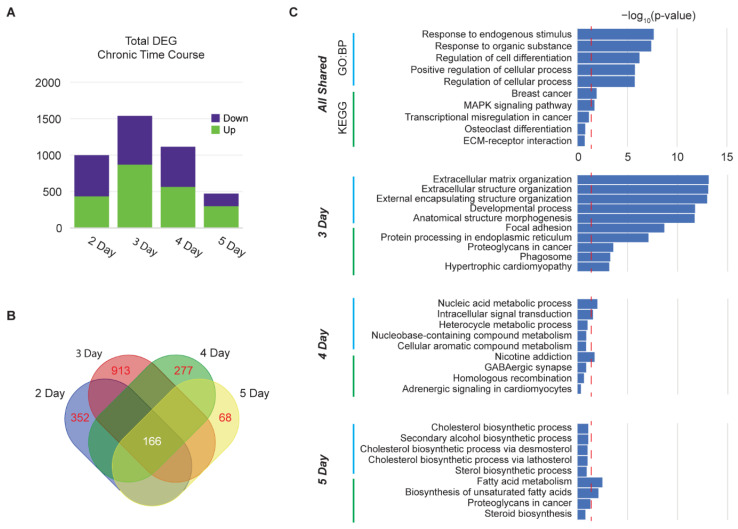
Time course of chronic dopamine administration reveals peak in DEGs at day 3 and desensitization at day 5. (**A**) Total number of DEGs from RNA-seq of DIV50 hMSN-like cells 1 h after 2, 3, 4, or 5 days of daily dosing of 1 mM dopamine. (**B**) Venn diagrams showing shared (white) and unique (red) numbers of DEGs between hMSN-like cells dosed with dopamine for 2–5 days. (**C**) Gene ontology biological processes and KEGG pathways for shared and unique genes from hMSN-like cells dosed with dopamine for 2–5 days. Significance is represented by Log_10_-transformed *p*-values with dotted red line indicating *p*-value of 0.05. In all cases, data were obtained from RNA-seq of H9 hMSN-like cells. DEGs were identified by FDR *p*-value < 0.05. Differential expression was performed against ascorbic acid vehicle control group using the Wald test. *n* = 2–3 independent replicates.

We observed desensitization in hMSN-like cells after 5 days of chronic dopamine dosing, similar to the length of administration in rodent models [1,14]. We next asked what the kinetics of gene expression changes and (de)sensitization were over time. We dosed hMSN-like cells with 1 mM dopamine once a day for five days and performed RNA-seq 1 h (DIV50) after each dose (Figure 4A and Figure S4, Dataset S6). We calculated the total number of DEGs over time and observed a peak on the third day of chronic dosing, which then dropped. We did observe a common set of genes that were differentially expressed at all time points (Figure 4B) and enriched for biological process GOs related to responses to stimuli and KEGG pathways such as MAPK signaling (Figure 4C). MAPK/ERK responses were observed prior to day 5, in agreement with our prior functional enrichment analysis of desensitized genes. However, despite this commonality, intriguingly, the nature of the response each day dynamically shifted. Genes unique to day 3 were related to the extracellular matrix organization and cell adhesion or morphogenesis. This then shifted, where day 4 was enriched for the nicotine addiction KEGG pathway, a pathway associated with nicotine-induced alterations in glutamatergic and GABAergic receptors (e.g., *GRIN1* and *GABRR2*) on GABAergic MSNs [52]. By day 5, the expression profile had shifted yet again to cholesterol and fatty acid metabolism. It is notable that the number of DEGs peaked at day 3, with many genes not activating or repressing until multiple doses of dopamine were administered.

### 3.5. hMSN-like Cells Capture Some Features of Dopamine Receptor Cross-Interactions

**Figure 5:**
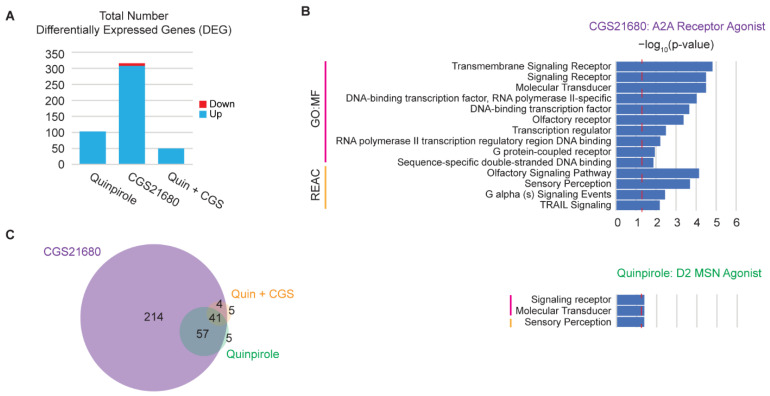
hMSN-like cells capture some features of dopamine receptor cross-interactions. (**A**) Total number of DEGs from RNA-seq of DIV45 hMSN-like cells dosed with receptor agonists. (**B**) Gene ontology molecular functions and reactome pathways for DEGs from hMSN-like cells dosed with ADORA2A agonist CGS21680 and D2-like receptor agonist quinpirole. Dotted red line indicates *p*-value of 0.05. (**C**) Venn diagram showing shared and unique numbers of DEGs for DIV45 hMSN-like cells dosed with agonists. In all cases, data were obtained from RNA-seq of DIV45 H9 hMSN-like cells. DEGs were identified by FDR *p*-value < 0.05. Differential expression was performed against a water (vehicle) control group using the Wald test. *n* = 3 independent replicates.

hMSN-like cells respond acutely to dopamine and exhibit desensitization to repeated exposures. However, one of the enduring challenges for MSN biology in rodents and humans is that MSNs exist as complex subtypes with multiple distinct receptors that may be expressed in many combinations [53,54,55]. Furthermore, these receptors can have complex downstream cross interactions. For example, D2-like receptor expression is normally associated with ADORA2A expression in the same MSNs in vivo [55]. Furthermore, D2-like receptors inhibit the activity of adenylyl cyclase downstream of ADORA2A [53,54,55]. We directly tested whether this crosstalk interaction between D2-like receptors and ADORA2A was maintained in human cells. We acutely and separately dosed DIV45 hMSN-like cells with the D2-like receptor agonist quinpirole and ADORA2A agonist CGS21680 (Figure 5 and Figure S5, Dataset S7). DEGs were the most abundant after dosage with CGS21680 (Figure 5A). CGS21680-induced DEGs were enriched for molecular function GO and reactome pathways related to G protein-coupled receptor signaling (Figure 5B), as expected for ADORA2A binding [53]. More interesting than the effects of individual agonists was when quinpirole and CGS21680 were added together. DEGs induced by quinpirole alone largely overlapped with those induced by CGS21680 alone (Figure 5C). However, preincubation with quinpirole an hour before the CGS21680 dosage negated the significant differential expression induced by CGS21680. This matches much of what is understood about D2-like receptors and ADORA2A. This result is also in alignment with previously studied interactions between the adenosine and dopamine systems [56]. This cross-interaction suggests that hMSN-like cells may be capable of capturing complex MSN signaling.

## 4. Discussion

New experimental models are needed to better understand the complex human-specific responses to dynamically variable perturbations. In this study, we highlighted the ability of hMSN-like cells to capture many simple gene expression responses as well as the more complex dynamic and signaling properties of the cells. These included gene desensitization in response to chronic dopamine exposure and receptor crosstalk in response to agonists of dopamine receptor subtypes. However, further development will be needed to improve these models, in particular, to improve the homogeneity and purity of hMSN-like cultures and to connect observed responses to physiological contexts. Identifying the similarities and differences to rodent models may also identify areas for improvement in hMSN-like models.

We showed that hMSN-like cells exhibited gene desensitization to a wide range of concentrations of dopamine. We also found that desensitized immediate early genes in hMSN-like cells, such as *FOS*, *FOSB*, *JUNB*, and *DUSP14*, matched those found in prior rat studies [1,57]. We also found that highly desensitized genes were associated with signaling through ERK1/2, a phenomenon also observed in the mice striatum in response to drugs of abuse [58]. Additionally, we described how the number of DEGs peaked at 3 days of chronic dopamine exposure and were functionally enriched at that point for processes thought to underlie neuroadaptations in substance use disorders. Specifically, alterations in the extracellular matrix organization may relate to the synaptic modifications observed after chronic cocaine exposure in mice [59]. These processes shifted to metabolic-related ontologies and pathways, along with a reduction in the total number of DEGs, after day 3. Finally, we showed that the D2-like receptor agonist quinpirole and the ADORA2A agonist CGS21680 induced DEGs. In addition, DEGs induced by CGS21680 were inhibited by quinpirole preincubation. Thus, D2-like receptors function as expected in hMSN-like cultures, and complex cross-interactions between receptors were observed. Overall, we observed overlap in many aspects of acute and chronic transcriptional responses and dynamics between hMSN-like cells and rodent MSNs. Understanding the transcriptional dynamics in hMSN-like cells may improve our understanding of the role specific genes play in the development of addictive behaviors in humans.

Despite many similarities, many differences also exist between the responses of hMSN-like cultures and rodent primary MSNs. Importantly, heterogeneity is observed in hMSN-like cells. For example, we observed the expression of genes characteristic of medial ganglionic eminence interneurons when MSNs should express only lateral ganglionic eminence genes [17]. This is partly due to the fact that differentiated culture systems in general do not generate pure populations of cells [60]. Furthermore, they may not be able to differentiate stem cells into fully mature, subtype-specific neurons, with neurons in development co-expressing mixtures of markers not observed in adult neurons [61]. It is important to also note that this heterogeneity may contribute to the differential responses observed in hMSN-like cells compared to rodent MSNs. For example, there were many more downregulated DEGs due to acute dopamine exposure observed in this study compared to what has previously been reported in rodents [40]. Furthermore, chronic dopamine dosage led to fewer DEGs in hMSN-like cells compared to more DEGs after chronic cocaine dosage in rats [1]. These differences could arise due to species-specific differences, heterogeneity, as well as experimental differences. For example, although we time-matched dosages and RNA extractions in most experiments, when comparing acute to chronic conditions, they cannot both be simultaneously matched. One additional experimental consideration is how dopamine dosing is performed in this study, where whole-cell incubation of dopamine does not necessarily replicate in vivo exposures. In vivo, dopamine exposure occurs at synapses on the length scale of tens of nanometers and time scale of microseconds [36]. However, given the rapid autooxidation rate of dopamine in vitro, higher concentrations of dopamine are potentially required to bind dopamine receptors despite precautions to maintain stability in acidic aqueous solutions [62]. A deviation in hMSN-like culture and rodent striatal MSN responses in culture may also be due to different distributions of MSN subtypes and precursors. For example, *FOS* expression in predominantly D2-like hMSN-like cells may be partly due to the natural lack of dopamine in basal hMSN-like culture media [17], as dopamine depletion in the globus pallidus has been shown to lead to *FOS* expression in response to quinpirole [63]. Additionally, the expression of dopamine receptor heteromers, which are more highly expressed in neonatal striatal neurons than in adult MSNs, may lead to unexpected transcriptional dynamics not seen in vivo [64]. hMSN-like cells may also not compare well with adult rodent studies because hMSN-like cells tend to lack functional maturity when compared to adult rodent neurons [61,65]. The lack of noticeable spines, characteristic of in vivo MSNs, also highlights the relative immaturity of hMSN-like cells [66]. Longer experiments may provide additional time for maturation but face practical experimental challenges. Functional maturation may also be due to the lack of other relevant cell types that MSNs reside with in vivo such as astrocytes, glutamatergic neurons, and dopaminergic neurons.

Future work could aim to improve the functional maturity and other phenotypes of hMSN-like cells that are prominently seen in primary rodent cultures. Prior work using primary rodent cultures has shown enhancements in functional characteristics such as increased dendritic spine density and spontaneous action potentials after cocultures with cortical neurons and astrocytes, respectively [67,68]. Similarly, cocultures between hMSN-like cells and other physiologically relevant human cell types may enhance the functional characteristics. Three-dimensional models of hMSN-like cells such as cerebral organoids could also potentially yield more mature neurons. In addition to improving neuronal maturation, new protocols that could either create or pan for purer populations of MSN subtypes would provide improved reductionist control to deconvolve the biology of MSNs. In addition, many protocols already exist to differentiate human stem cells to DARPP32+ striatal neurons [18,69] and it would be advantageous for the field to identify the differences in heterogeneity produced using each protocol using single-cell sequencing technologies. Future work could also include the purification of target cell types by tagging the *DARPP32* gene with a fluorescent reporter and sorting via flow cytometry [70]. Finally, it would be interesting to investigate the post-translational and epigenetic changes in histone modifications that may underlie the mechanisms of desensitization, whether these changes are observed in hMSN-like cells in response to chronic dopamine administration as they are observed in rodents, as well as expand to other dosing regimens to investigate transcriptional or epigenetic changes in experiments mimicking withdrawal [71,72]. For example, we observed transcriptional desensitization of the ERK pathway genes; determining if post-translational phosphorylation of ERK1/2 is also desensitized would be of interest mechanistically. The work here highlights the promise as well as current limitations of stem cell-derived hMSN-like cells as a model to study complex responses to substances of abuse and other diverse perturbations in humans.

## Data Availability

Publicly available datasets were analyzed in this study. This data can be found here: [https://www.ncbi.nlm.nih.gov/geo/query/acc.cgi?acc=GSE195492 (accessed on 25 March 2022)].

## Supplementary Materials

The following supporting information can be downloaded at: https://www.mdpi.com/article/10.3390/cells11091411/s1, Figure S1: QRT-PCR of target genes as compared to *GUSB*; Figure S2: FOSB immunostaining highlights differential responses to acute and chronic dopamine; Figure S3: Heatmaps and principal component analysis of acute and chronic dopamine-dosed hMSN-like cells; Figure S4: Heatmaps and principal component analysis of chronic dopamine time-course-dosed hMSN-like cells; Figure S5: Heatmaps and principal component analysis of agonist-dosed hMSN-like cells; Table S1: Primary Antibodies; Table S2: RT-qPCR Primers; Dataset S1: Overlapping and unique differentially expressed genes between 1 μM and 1 mM acute dopamine conditions one hour after an acute dosage (Acute 1 h); Dataset S2: Overlapping and unique differentially expressed genes between 1 μM and 1 mM acute dopamine conditions twenty four hours after an acute dosage (Acute 24 h); Dataset S3: Overlapping and unique differentially expressed genes between 1 μM and 1 mM chronic dopamine conditions one hour after a chronic dosage (Chronic 1 h); Dataset S4: Overlapping and unique differentially expressed genes between 1 μM and 1 mM chronic dopamine conditions twenty four hours after a chronic dosage (Chronic 24 h); Dataset S5: Overlapping and unique desensitized differentially expressed genes between 1 μM and 1 mM dopamine conditions one hour after dosage (1 h); Dataset S6: Overlapping and unique differentially expressed genes between 2-day through 5-day chronic dopamine dosed hMSN-like cells one hour after dosage; Dataset S7: Overlapping and total differentially expressed genes between agonist dosed conditions 1 h after dosage as compared to water control.

## References

[1] Maze I., Iii H.E.C., Dietz D.M., Laplant Q., Renthal W., Russo S.J., Mechanic M., Mouzon E., Neve R.L., Stephen J. (2010). Essential Role of the Histone Methyltransferase G9a in Cocaine Induced Plasticity. Science.

[2] Renthal W., Carle T.L., Maze I., Covington H.E., Truong H.-T., Alibhai I., Kumar A., Montgomery R.L., Olson E.N., Nestler E.J. (2008). ΔFosB Mediates Epigenetic Desensitization of the C-Fos Gene After Chronic Amphetamine Exposure. J. Neurosci..

[3] Rusconi F., Battaglioli E. (2018). Acute Stress-Induced Epigenetic Modulations and Their Potential Protective Role Toward Depression. Front. Mol. Neurosci..

[4] Nestler E.J., Barrot M., Self D.W. (2001). FosB: A Sustained Molecular Switch for Addiction. Proc. Natl. Acad. Sci. USA.

[5] Tang T.-S., Slow E., Lupu V., Stavrovskaya I.G., Sugimori M., Llinás R., Kristal B.S., Hayden M.R., Bezprozvanny I. (2005). Disturbed Ca^2+^ Signaling and Apoptosis of Medium Spiny Neurons in Huntington’s Disease. Proc. Natl. Acad. Sci. USA.

[6] Deutch A.Y., Colbran R.J., Winder D.J. (2007). Striatal Plasticity and Medium Spiny Neuron Dendritic Remodeling in Parkinsonism. Parkinsonism Relat. Disord..

[7] Chandra R., Lobo M.K. (2017). Beyond Neuronal Activity Markers: Select Immediate Early Genes in Striatal Neuron Subtypes Functionally Mediate Psychostimulant Addiction. Front. Behav. Neurosci..

[8] Le Cann K., Foerster A., Rösseler C., Erickson A., Hautvast P., Giesselmann S., Pensold D., Kurth I., Rothermel M., Mattis V.B. (2021). The Difficulty to Model Huntington’s Disease in Vitro Using Striatal Medium Spiny Neurons Differentiated from Human Induced Pluripotent Stem Cells. Sci. Rep..

[9] Lissek T., Andrianarivelo A., Saint-Jour E., Allichon M.-C., Bauersachs H.G., Nassar M., Piette C., Pruunsild P., Tan Y.-W., Forget B. (2021). Npas4 Regulates Medium Spiny Neuron Physiology and Gates Cocaine-Induced Hyperlocomotion. EMBO Rep..

[10] Yuen J., Goyal A., Rusheen A.E., Kouzani A.Z., Berk M., Kim J.H., Tye S.J., Blaha C.D., Bennet K.E., Jang D.-P. (2021). Cocaine-Induced Changes in Tonic Dopamine Concentrations Measured Using Multiple-Cyclic Square Wave Voltammetry in Vivo. Front. Pharmacol..

[11] Heien M.L.A.V., Khan A.S., Ariansen J.L., Cheer J.F., Phillips P.E.M., Wassum K.M., Wightman R.M. (2005). Real-Time Measurement of Dopamine Fluctuations after Cocaine in the Brain of Behaving Rats. Proc. Natl. Acad. Sci. USA.

[12] Mishra A., Singh S., Shukla S. (2018). Physiological and Functional Basis of Dopamine Receptors and Their Role in Neurogenesis: Possible Implication for Parkinson’s Disease. J. Exp. Neurosci..

[13] Diana M. (2011). The Dopamine Hypothesis of Drug Addiction and Its Potential Therapeutic Value. Front. Psychiatry.

[14] Kumar A., Choi K.H., Renthal W., Tsankova N.M., Theobald D.E.H., Truong H.T., Russo S.J., LaPlant Q., Sasaki T.S., Whistler K.N. (2005). Chromatin Remodeling Is a Key Mechanism Underlying Cocaine-Induced Plasticity in Striatum. Neuron.

[15] Nestler E. (2005). The Neurobiology of Cocaine Addiction. Sci. Pract. Perspect..

[16] Dudman J.T., Gerfen C.R. (2015). Chapter 17—The Basal Ganglia. Paxinos, G.B.T.-T.R.N.S.

[17] Arber C., Precious S.V., Cambray S., Risner-Janiczek J.R., Kelly C., Noakes Z., Fjodorova M., Heuer A., Ungless M.A., Rodriguez T.A. (2015). Activin A Directs Striatal Projection Neuron Differentiation of Human Pluripotent Stem Cells. Development.

[18] Golas M.M. (2018). Human Cellular Models of Medium Spiny Neuron Development and Huntington Disease. Life Sci..

[19] Carri A.D., Onorati M., Lelos M.J., Castiglioni V., Faedo A., Menon R., Camnasio S., Vuono R., Spaiardi P., Talpo F. (2013). Developmentally Coordinated Extrinsic Signals Drive Human Pluripotent Stem Cell Differentiation toward Authentic DARPP-32+ Medium-Sized Spiny Neurons. Development.

[20] Hunt C.P.J., Pouton C.W., Haynes J.M. (2017). Characterising the Developmental Profile of Human Embryonic Stem Cell-Derived Medium Spiny Neuron Progenitors and Assessing Mature Neuron Function Using a CRISPR-Generated Human DARPP-32WT/EGFP-AMPreporter Line. Neurochem. Int..

[21] Iannielli A., Ugolini G.S., Cordiglieri C., Bido S., Rubio A., Colasante G., Valtorta M., Cabassi T., Rasponi M., Broccoli V. (2019). Reconstitution of the Human Nigro-Striatal Pathway on-a-Chip Reveals OPA1-Dependent Mitochondrial Defects and Loss of Dopaminergic Synapses. Cell Rep..

[22] Grigor’eva E.V., Malankhanova T.B., Surumbayeva A., Pavlova S.V., Minina J.M., Kizilova E.A., Suldina L.A., Morozova K.N., Kiseleva E., Sorokoumov E.D. (2020). Generation of GABAergic Striatal Neurons by a Novel IPSC Differentiation Protocol Enabling Scalability and Cryopreservation of Progenitor Cells. Cytotechnology.

[23] Schindelin J., Arganda-Carreras I., Frise E., Kaynig V., Longair M., Pietzsch T., Preibisch S., Rueden C., Saalfeld S., Schmid B. (2012). Fiji: An Open-Source Platform for Biological-Image Analysis. Nat. Methods.

[24] Sen D., Voulgaropoulos A., Drobna Z., Keung A.J. (2020). Human Cerebral Organoids Reveal Early Spatiotemporal Dynamics and Pharmacological Responses of UBE3A. Stem Cell Rep..

[25] Untergasser A., Cutcutache I., Koressaar T., Ye J., Faircloth B.C., Remm M., Rozen S.G. (2012). Primer3--New Capabilities and Interfaces. Nucleic Acids Res..

[26] Krämer A., Green J., Pollard J., Tugendreich S. (2014). Causal Analysis Approaches in Ingenuity Pathway Analysis. Bioinformatics.

[27] Raudvere U., Kolberg L., Kuzmin I., Arak T., Adler P., Peterson H., Vilo J. (2019). G:Profiler: A Web Server for Functional Enrichment Analysis and Conversions of Gene Lists (2019 Update). Nucleic Acids Res..

[28] Allaire J. (2012). Rstudio: Integrated Development Environment for R. https://www.r-project.org/conferences/useR-2011/abstracts/180111-allairejj.pdf.

[29] Hulsen T. (2021). BioVenn—An R and Python Package for the Comparison and Visualization of Biological Lists Using Area-Proportional Venn Diagrams. Data Sci..

[30] VIB-UGENT for Plant Systems Biology Calculate and Draw Custom Venn Diagrams. http://bioinformatics.psb.ugent.be/webtools/Venn/.

[31] Wickham H. (2016). Ggplot2: Elegant Graphics for Data Analysis.

[32] Gokce O., Stanley G.M., Treutlein B., Neff N.F., Camp J.G., Malenka R.C., Rothwell P.E., Fuccillo M.V., Südhof T.C., Quake S.R. (2016). Cellular Taxonomy of the Mouse Striatum as Revealed by Single-Cell RNA-Seq. Cell Rep..

[33] Straccia M., Barriga G.G.D., Sanders P., Bombau G., Carrere J., Mairal P.B., Vinh N.N., Yung S., Kelly C.M., Svendsen C.N. (2015). Quantitative High-Throughput Gene Expression Profiling of Human Striatal Development to Screen Stem Cell-Derived Medium Spiny Neurons. Mol. Ther. -Methods Clin. Dev..

[34] Benarroch E.E. (2012). Effects of Acetylcholine in the Striatum. Recent Insights and Therapeutic Implications. Neurology.

[35] Garris P.A., Ciolkowski E.L., Pastore P., Wightman R.M. (1994). Efflux of Dopamine from the Synaptic Cleft in the Nucleus Accumbens of the Rat Brain. J. Neurosci..

[36] Liu C., Goel P., Kaeser P.S. (2021). Spatial and Temporal Scales of Dopamine Transmission. Nat. Rev. Neurosci..

[37] Marcott P.F., Mamaligas A.A., Ford C.P. (2014). Phasic Dopamine Release Drives Rapid Activation of Striatal D2-Receptors. Neuron.

[38] Ofori S., Bugnon O., Schorderet M. (1993). Agonist-Induced Desensitization of Dopamine D-1 Receptors in Bovine Retina and Rat Striatum. J. Pharmacol. Exp. Ther..

[39] Memo M., Lovenberg W., Hanbauer I. (1982). Agonist-Induced Subsensitivity of Adenylate Cyclase Coupled with a Dopamine Receptor in Slices from Rat Corpus Striatum. Proc. Natl. Acad. Sci. USA.

[40] Savell K.E., Tuscher J.J., Zipperly M.E., Duke C.G., Phillips R.A., Bauman A.J., Thukral S., Sultan F.A., Goska N.A., Ianov L. (2020). A Dopamine-Induced Gene Expression Signature Regulates Neuronal Function and Cocaine Response. Sci. Adv..

[41] Scimemi A., Beato M. (2009). Determining the Neurotransmitter Concentration Profile at Active Synapses. Mol. Neurobiol..

[42] Berke J.D., Paletzki R.F., Aronson G.J., Hyman S.E., Gerfen C.R. (1998). A Complex Program of Striatal Gene Expression Induced by Dopaminergic Stimulation. J. Neurosci..

[43] Attisano L., Wrana J.L. (2013). Signal Integration in TGF-β, WNT, and Hippo Pathways. F1000Prime Rep..

[44] Weissenrieder J.S., Neighbors J.D., Mailman R.B., Hohl R.J. (2019). Cancer and the Dopamine D(2) Receptor: A Pharmacological Perspective. J. Pharmacol. Exp. Ther..

[45] Sobczuk P., Łomiak M., Cudnoch-Jędrzejewska A. (2020). Dopamine D1 Receptor in Cancer. Cancers.

[46] Zipperly M.E., Sultan F.A., Graham G.-E., Brane A.C., Simpkins N.A., Carullo N.V.N., Ianov L., Day J.J. (2021). Regulation of Dopamine-Dependent Transcription and Cocaine Action by Gadd45b. Neuropsychopharmacology.

[47] Ethell I.M., Ethell D.W. (2007). Matrix Metalloproteinases in Brain Development and Remodeling: Synaptic Functions and Targets. J. Neurosci. Res..

[48] Heller E.A., Cates H.M., Peña C.J., Sun H., Shao N., Feng J., Golden S.A., Herman J.P., Walsh J.J., Mazei-Robison M. (2014). Locus-Specific Epigenetic Remodeling Controls Addiction- and Depression-Related Behaviors. Nat. Neurosci..

[49] Vialou V., Maze I., Renthal W., LaPlant Q.C., Watts E.L., Mouzon E., Ghose S., Tamminga C.A., Nestler E.J. (2010). Serum Response Factor Promotes Resilience to Chronic Social Stress through the Induction of ΔFosB. J. Neurosci..

[50] Nennig S.E., Schank J.R. (2017). The Role of NFkB in Drug Addiction: Beyond Inflammation. Alcohol Alcohol..

[51] Cates H.M., Heller E.A., Lardner C.K., Purushothaman I., Peña C.J., Walker D.M., Cahill M.E., Neve R.L., Shen L., Bagot R.C. (2018). Transcription Factor E2F3a in Nucleus Accumbens Affects Cocaine Action via Transcription and Alternative Splicing. Biol. Psychiatry.

[52] Mansvelder H.D., McGehee D.S. (2002). Cellular and Synaptic Mechanisms of Nicotine Addiction. J. Neurobiol..

[53] Greengard P., Allen P.B., Nairn A.C. (1999). Beyond the Dopamine Receptor: The DARPP-32/Protein Phosphatase-1 Cascade. Neuron.

[54] Surmeier D.J., Ding J., Day M., Wang Z., Shen W. (2007). D1 and D2 Dopamine-Receptor Modulation of Striatal Glutamatergic Signaling in Striatal Medium Spiny Neurons. Trends Neurosci..

[55] Swapna I., Bondy B., Morikawa H. (2016). Differential Dopamine Regulation of Ca2+ Signaling and Its Timing Dependence in the Nucleus Accumbens. Cell Rep..

[56] Zhang X., Nagai T., Ahammad R.U., Kuroda K., Nakamuta S., Nakano T., Yukinawa N., Funahashi Y., Yamahashi Y., Amano M. (2019). Balance between Dopamine and Adenosine Signals Regulates the PKA/Rap1 Pathway in Striatal Medium Spiny Neurons. Neurochem. Int..

[57] Hope B., Kosofsky B., Hyman S.E., Nestler E.J. (1992). Regulation of Immediate Early Gene Expression and AP-1 Binding in the Rat Nucleus Accumbens by Chronic Cocaine. Proc. Natl. Acad. Sci. USA.

[58] Valjent E., Pascoli V., Svenningsson P., Paul S., Enslen H., Corvol J.-C., Stipanovich A., Caboche J., Lombroso P.J., Nairn A.C. (2005). Regulation of a Protein Phosphatase Cascade Allows Convergent Dopamine and Glutamate Signals to Activate ERK in the Striatum. Proc. Natl. Acad. Sci. USA.

[59] Zhang L., Huang L., Lu K., Liu Y., Tu G., Zhu M., Ying L., Zhao J., Liu N., Guo F. (2017). Cocaine-Induced Synaptic Structural Modification Is Differentially Regulated by Dopamine D1 and D3 Receptors-Mediated Signaling Pathways. Addict. Biol..

[60] Jerber J., Seaton D.D., Cuomo A.S.E., Kumasaka N., Haldane J., Steer J., Patel M., Pearce D., Andersson M., Bonder M.J. (2021). Population-Scale Single-Cell RNA-Seq Profiling across Dopaminergic Neuron Differentiation. Nat. Genet..

[61] Berry B.J., Smith A.S.T., Young J.E., Mack D.L. (2018). Advances and Current Challenges Associated with the Use of Human Induced Pluripotent Stem Cells in Modeling Neurodegenerative Disease. Cells Tissues Organs.

[62] Umek N., Geršak B., Vintar N., Šoštarič M., Mavri J. (2018). Dopamine Autoxidation Is Controlled by Acidic PH. Front. Mol. Neurosci..

[63] Robertson G.S., Vincent S.R., Fibiger H.C. (1992). D1 and D2 Dopamine Receptors Differentially Regulate C-Fos Expression in Striatonigral and Striatopallidal Neurons. Neuroscience.

[64] Perreault M.L., Hasbi A., Alijaniaram M., O’Dowd B.F., George S.R. (2012). Reduced Striatal Dopamine D1-D2 Receptor Heteromer Expression and Behavioural Subsensitivity in Juvenile Rats. Neuroscience.

[65] Qian X., Song H., Ming G. (2019). Brain Organoids: Advances, Applications and Challenges. Development.

[66] Lee K.-W., Kim Y., Kim A.M., Helmin K., Nairn A.C., Greengard P. (2006). Cocaine-Induced Dendritic Spine Formation in D1 and D2 Dopamine Receptor-Containing Medium Spiny Neurons in Nucleus Accumbens. Proc. Natl. Acad. Sci. USA.

[67] Penrod R.D., Campagna J., Panneck T., Preese L., Lanier L.M. (2015). The Presence of Cortical Neurons in Striatal-Cortical Co-Cultures Alters the Effects of Dopamine and BDNF on Medium Spiny Neuron Dendritic Development. Front. Cell. Neurosci..

[68] Aebersold M.J., Thompson-Steckel G., Joutang A., Schneider M., Burchert C., Forró C., Weydert S., Han H., Vörös J. (2018). Simple and Inexpensive Paper-Based Astrocyte Co-Culture to Improve Survival of Low-Density Neuronal Networks. Front. Neurosci..

[69] Delli Carri A., Onorati M., Castiglioni V., Faedo A., Camnasio S., Toselli M., Biella G., Cattaneo E. (2013). Human Pluripotent Stem Cell Differentiation into Authentic Striatal Projection Neurons. Stem Cell Rev. Reports.

[70] Cruz-Santos M., Cardo L.F., Li M. (2022). A Novel LHX6 Reporter Cell Line for Tracking Human IPSC-Derived Cortical Interneurons. Cells.

[71] Renthal W., Nestler E. (2008). Epigenetic Mechanisms in Drug Addiction. Mol. Med..

[72] Eipper-Mains J.E., Kiraly D.D., Duff M.O., Horowitz M.J., McManus C.J., Eipper B.A., Graveley B.R., Mains R.E. (2013). Effects of Cocaine and Withdrawal on the Mouse Nucleus Accumbens Transcriptome. Genes, Brain Behav..

