# Supplementary material for: Human Pluripotent Stem Cell-Derived Medium Spiny Neuron-like Cells Exhibit Gene Desensitization
Source: Cells. 2022 Apr 21;11(9):1411. doi: 10.3390/cells11091411 (PMC9100557; doi:10.3390/cells11091411)
Supplement: Supplementary file 1 [file cells-11-01411-s001.zip › cells-1674018-supplementary/Supporting Information/Supporting Figures and Tables.pdf]

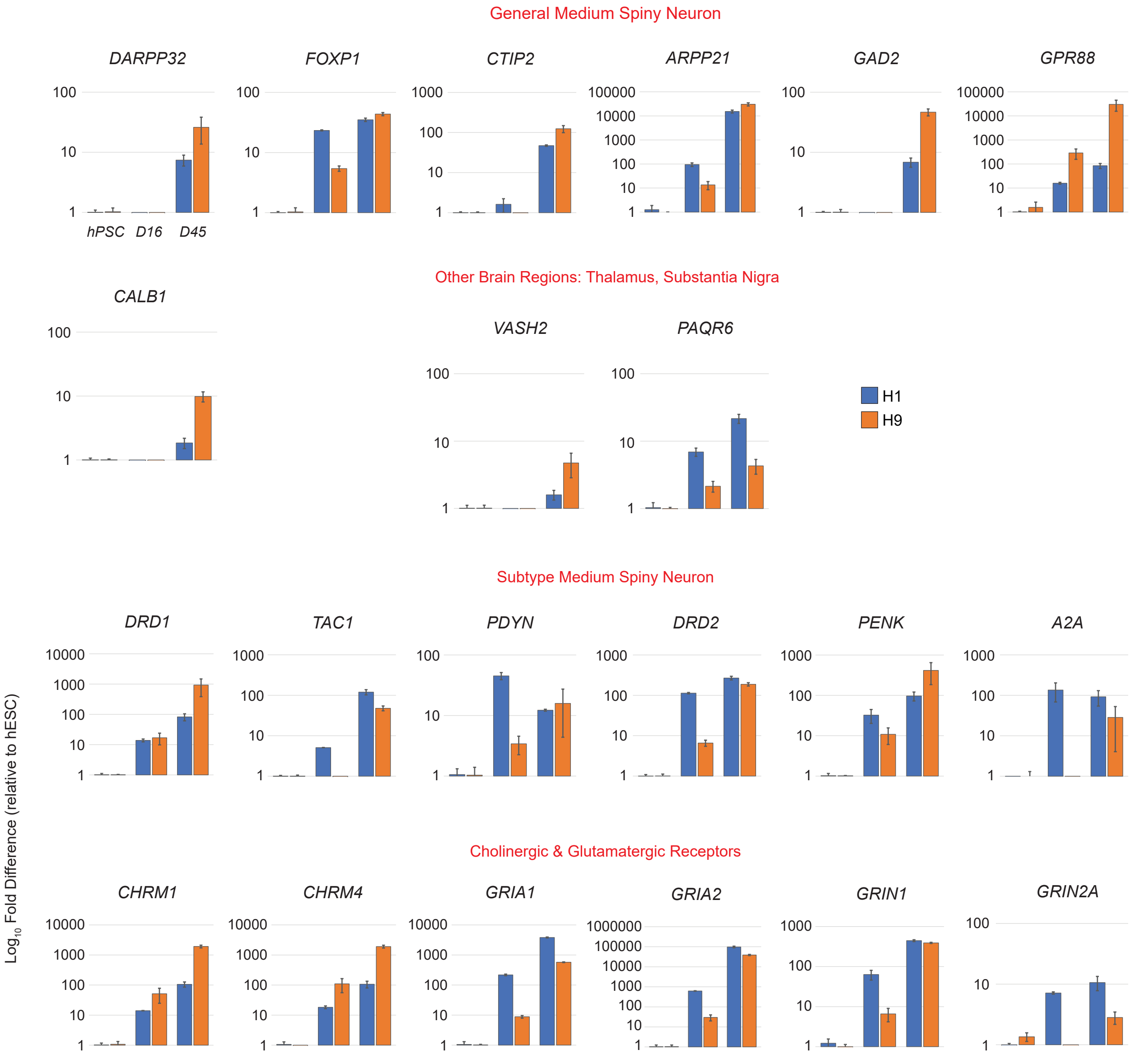

Figure S1. QRT-PCR of Target Genes as Compared to GUSB. QRT-PCR of RNA isolated from H1 (blue) and H9 (orange) hPSCs and hPSCs differentiated into hMSN-like cells at DIV 16 (D16) and DIV 45 (D45), for genes of interest. Values were normalized to GUSB mRNA levels in the same samples and expressed as normalized Log_{10} fold change values in hMSN-like versus hPSC cells. Gene categories are labeled in red. n = 3-4 independent replicates & 2 technical replicates. Error bars = Standard error.

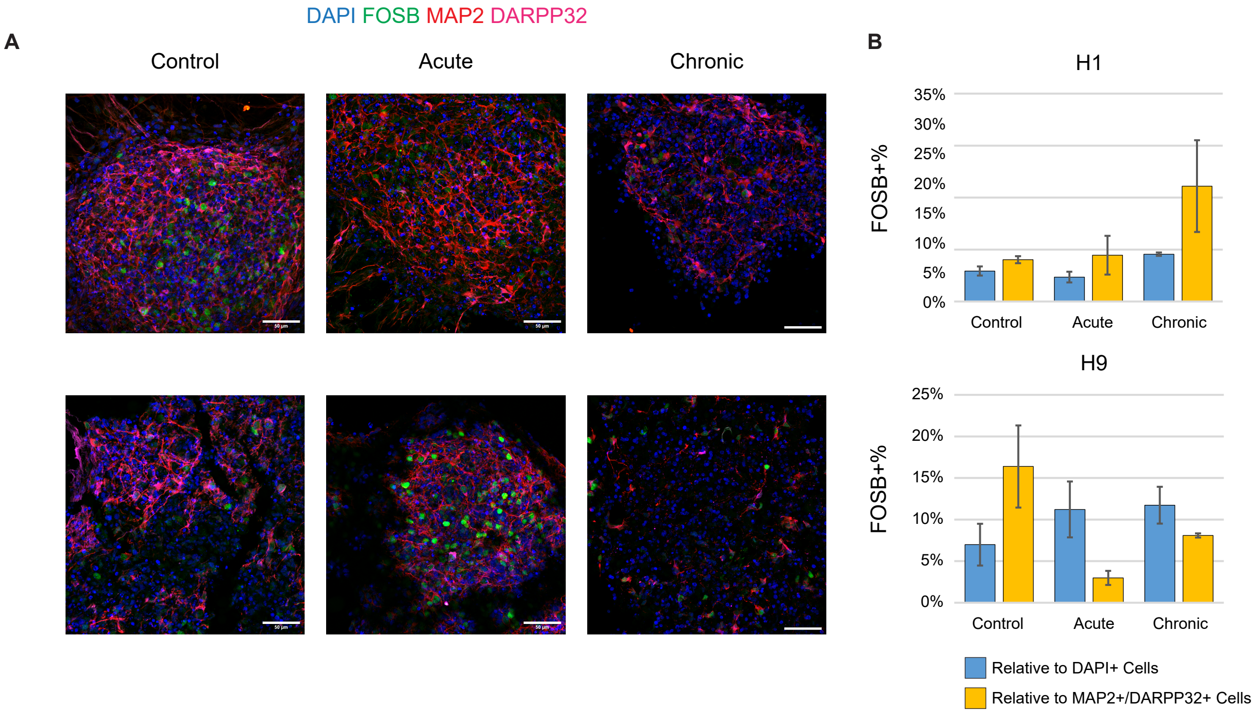

Figure S2. FOSB Immunostaining highlights differential responses to acute and chronic dopamine. (A) Representative immunostaining images of FOSB+ cells after control 100 μM Ascorbic Acid, 1 mM Acute and 1 mM Chronic dopamine doses on DIV45 for H1 (top) and H9 (bottom) hMSN-like cells. (B) Quantification of immunostained samples. Error bars are standard deviation. n = 2-3 independent replicates. Scale bar = 50 μm.

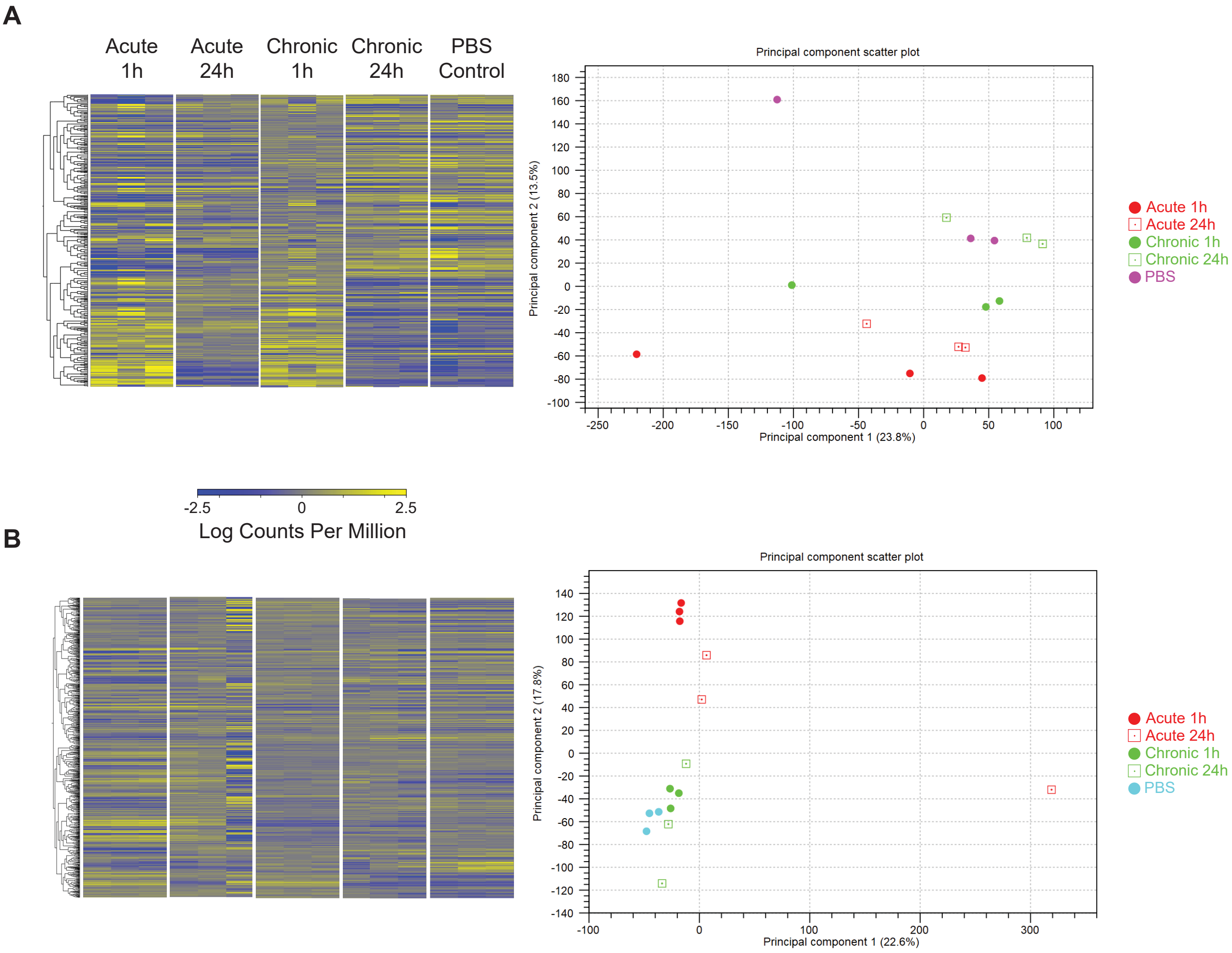

Figure S3. Heatmaps and Principal Component Analysis of Acute and Chronic Dopa-mine-dosed hMSN-like cells. Related to Figures 2 and 3. (A) 1 μM acute (DIV45)/chronic (DIV50) dopamine dosing regimen and a (B) 1 mM acute/ chronic dopamine dosing regimen. (Left) Heatmap visualization of normalized gene counts after hiererachical clustering across conditions. Each column is one replicate of the labeled condition. n = 3 independent replicates. (Right) Principal Component Anal-ysis (PCA). Analysis performed in CLC Workbench.

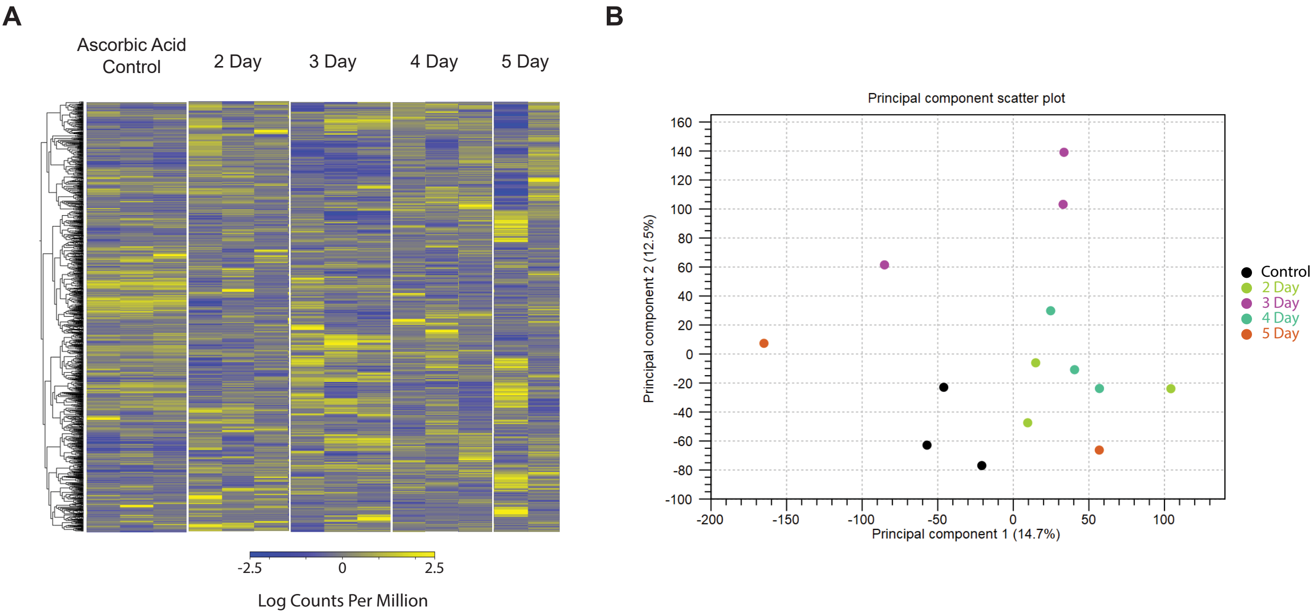

Figure S4. Heatmaps and Principal Component Analysis of Chronic Dopamine time course-dosed (DIV50) hMSN-like cells. Related to Figure 4. (A) Heatmap visualiza-tion of normalized gene counts after hiererachical clustering across conditions. Each column is one replicate of the labeled condition. n = 2-3 independent repli-cates. (B) Principal Component Analysis (PCA). Analysis performed in CLC Work-bench.

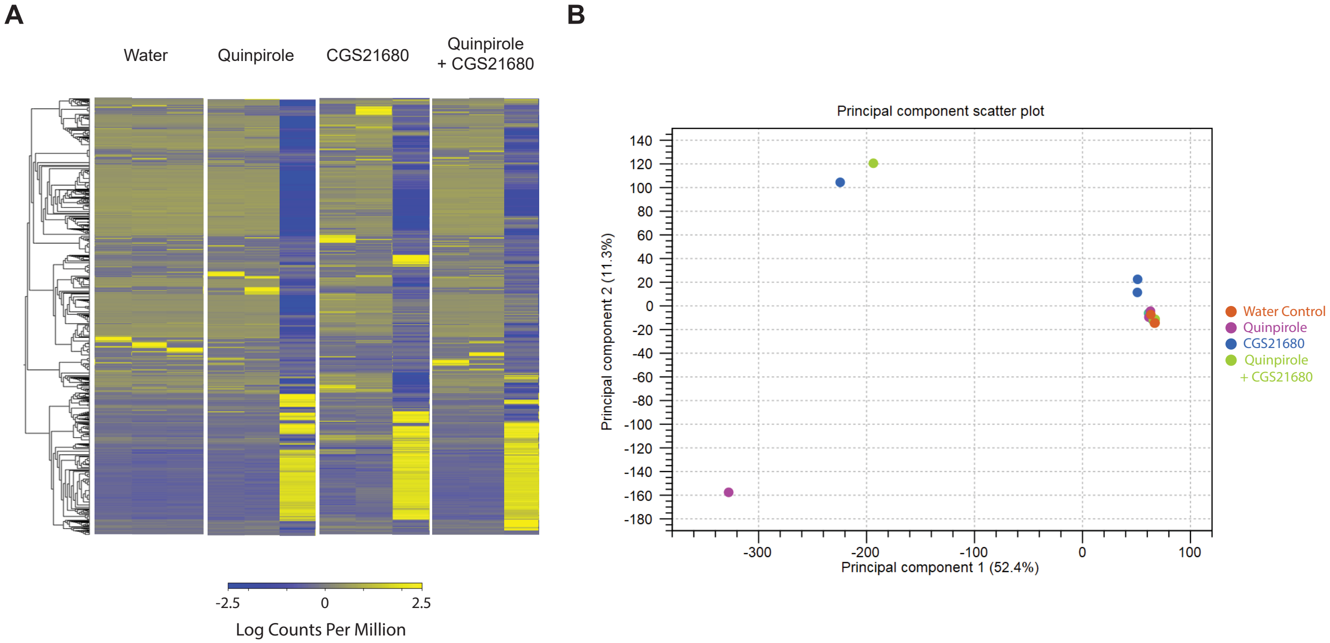

Figure S5. Heatmaps and Principal Component Analysis of DIV45 Agonist-dosed hMSN-like cells. Related to Figure 5. (A) Heatmap visualization of normalized gene counts after hiererachical clustering across conditions. Each column is one repli-cate of the labeled condition. n = 3 independent replicates. (B) Principal Compo-nent Analysis (PCA). Analysis performed in CLC Workbench.

Table S1. Primary antibodies. Related to Figure 1.

Antigen	Host	Supplier	Cat. No.	RRID	Dilution
DARPP32	Goat	R&D Systems	AF6259	AB_10641854	1:13
MAP2	Mouse	Millipore Sigma	M1406	AB_477171	1:375
GFP	Chicken	Abcam	AB13970	AB_300798	1:500

Table S2. QRT-PCR Primers. Related to Figure 1.

Gene Symbol	Gene ID	Forward Primer	Reverse Primer
DRD1	1812	AGGTATTGGGCTATCTCCAGC	AGATGAGTACAGACAAGGTCCAT
DRD2	1813	CTCTTCGGACTCAATAACGCAG	GACGATGGAGGAGTAGACCAC
FOXP1	27086	AGGCCACAAAAGATCAGTGG	GCCATTGAAGCCTGTAAAGC
GAD2	2572	CTATGACACTGGAGACAAGGC	CAAACATTTATCAACATGCGCTTC
TAC1	6863	GTACGACAGCGACCAGATCA	AGCCTTTAACAGGGCCACTT
PDYN	5173	GGTGCTCCTTGTGTGCT	CATCTCTCCCATTCCTCAGA
A2A	135	AGGCAGCAAGAACCTTTCAA	CTAAGGAGCTCCACGTCTGG
PENK	5179	GCTGTCCAAACCAGAGCTTC	TCTGGCTCCATGGGATAAAG
CTIP2	64919	CAACCCGCAGCACTTGTC	CCTCGTCTTCTTCGAGGATGG
ARPP21	10777	GTGCAAAGCGTGATGGTTTCC	CCTTGACCTGCCTGGTTAGG
CALB1	793	ATCAGGACGGCAATGGATAC	TAAGAGCAAGATCCGTTCGG
GPR88	54112	ATCCCGGTGTCACTCCTGTAT	CACGAGATAGATGACCATGCC
OPRM1	4988	CAGCCATTGGTCTTCCTGTA	TCAGCAGGTTTTCCCAGTAC
RARB	5915	TCCGAAAAGCTCACCAGGAAA	GGCCAGTTCACTGAATTTGTCC
CHRM1	1128	CTCTATACCACGTACCTGCTCA	CCGAGTCACGGAGAAGTAGC
CHRM4	1132	AGGACACTTCCAATGAGTCCA	TGTCTGCTTCGTCACAATCTG
GRIA1	2890	TGCTTTGTCGCAACTCACAGA	GGCATAGACTCCTTTGGAGAAC
GRIA2	2891	CACCCCACATCGACAATTTGG	GACGTGGAGTGTTCCGCAA
GRIA3	2892	TCCGGGCGGTCTTCTTTTTAG	TCCACCTATGCTGATGGTGTT
GRIN1	2902	CTACCGCATACCCGTGCTG	GCATCATCTCAAACCACACGC
GRIN2A	2903	GTCCTTCTCCGACTGTGAGC	CCTTGCAGCATTTCTTCACA
GRIN2B	2904	TCTTCCTGGCCAGCTACACT	GGAACTTTTTGTCGCTCAGG
GUSB	2990	CAGCGTGGAGCAAGACAGTGG	AATACAGATAGGCAGGGCGTTCG
VASH2	79805	TTGGCAAAGCCTTCAATACC	GTAATTCTGGATCGCCTGGA
PAQR6	79957	CCTTCCCCTATGCCGCCTA	GGAGGACCTTACTGAGCCC
DARRP32	84152	TTGGAAAATCCAGAAAACCG	CTGGTAGAAGCCGGTGAGAG
FOSB	2354	AGCAGCAGCTAAATGCAGGA	TTCGTAGGGGATCTTGCAGC

FOS	2353	GGGGCAAGGTGGAACAGTTA	TCCTTCAGCAGGTTGGCAAT
DRD3	1814	TGGTAAACTCCTCGGTCTCCAGA	CAGAGATGCCATAGCCCAGAGG
DRD4	1815	AACTCCTTCATCGTGAGCCT	CGCACAGGTTGAAGATGGAG
DRD5	1816	TTGCTGAGTCTGTCTGGGAG	TAAAGGGAGCAGCACTGGAA
NKX2.1	2290	GGACGTGAGCAAGAACATGG	AACCAGATCTTGACCTGCGT
SST	7080	CTCCGTCAGTTTCTGCAGAA	TCAGGTTCCAGGGCATCATT
PVALB	6750	AAAGAGTGCGGATGATGTGAAG	ACCCCAATTTTGCCGTCCC
FOXG1	5816	CCCTGCCCTGTGAGTCTTTA	GGTTGGAAGAAGACCCCTGA
NR2F2	7026	GGAGAAGCTCAAGGCGCTGCA	CCTGCAAGCTTTCCACATGGG
